# Adoptive immunotherapy for gastric cancer using zoledronate-activated killer cells: A prospective observational study

**DOI:** 10.3892/mco.2021.2454

**Published:** 2021-11-25

**Authors:** Yoshiyuki Yamaguchi, Yousuke Katata, Fuminori Sano, Hiroaki Tanioka, Makoto Okawaki, Masahiro Yamamura, Takeshi Nagasaka

Mol Clin Oncol 13:Article no. 55, 2020; DOI: 10.3892/mco.2020.2125

Subsequently to the publication of this article, the authors wish to change the information that is presented in relation to the Research Ethics Committee and approval number for their prospective study, which is no longer accurated as reported. The final sentence in the ‘S*tudy design*’ subsection of the Patients and methods, as featured on p. 2 [‘This prospective study was reviewed about science and ethics and approved by the Research Ethics Committee of Kawasaki Medical School and Hospital (approval no. 240, UMIN000021797)]’ should be amended to the following: ‘**This prospective study was reviewed for its science and ethics and approved by the Certified Committee for Regenerative Medicine of Kawasaki Medical School Hospital (Committee number, NB6150002; protocol number, PC6150017; Japan Registry of Clinical Trials [jRCT] number, jRCTc060190032’.** Likewise, the first sentence in the ‘**Ethics approval and consent to participate**’ section of the Declarations on p. 7 should now read as follows: ‘The present study was reviewed and approved by the **Certified Committee for Regenerative Medicine of Kawasaki Medical School Hospital (Committee number, NB6150002; protocol number, PC6150017; Japan Registry of Clinical Trials [jRCT] number, jRCTc060190032’**.

The authors apologize to the readership of the Journal for any inconvenience caused.

